# *Saccharomyces cerevisiae* – Insects Association: Impacts, Biogeography, and Extent

**DOI:** 10.3389/fmicb.2020.01629

**Published:** 2020-07-14

**Authors:** Niccolo’ Meriggi, Monica Di Paola, Duccio Cavalieri, Irene Stefanini

**Affiliations:** ^1^Department of Biology, University of Florence, Florence, Italy; ^2^Department of Life Sciences and Systems Biology, University of Turin, Turin, Italy

**Keywords:** *Saccharomyces cerevisiae*, insect, yeast-insect association, biogeography, *Saccharomyces cerevisiae* evolution, *Saccharomyces cerevisiae* yeast ecology

## Abstract

Over the last few years, an increasing number of studies have reported the existence of an association between the budding yeast *Saccharomyces cerevisiae* and insects. The discovery of this relationship has called into question the hypothesis that *S. cerevisiae* is unable to survive in nature and that the presence of *S. cerevisiae* strains in natural specimens is the result of contamination from human-related environments. *S. cerevisiae* cells benefit from this association as they find in the insect intestine a shelter, but also a place where they can reproduce themselves through mating, the latter being an event otherwise rarely observed in natural environments. On the other hand, insects also take advantage in hosting *S. cerevisiae* as they rely on yeasts as nutriment to properly develop, to localize suitable food, and to enhance their immune system. Despite the relevance of this relationship on both yeast and insect ecology, we are still far from completely appreciating its extent and effects. It has been shown that other yeasts are able to colonize only one or a few insect species. Is it the same for *S. cerevisiae* cells or is this yeast able to associate with any insect? Similarly, is this association geographically or topographically limited in areas characterized by specific physical features? With this review, we recapitulate the nature of the *S. cerevisiae*-insect association, disclose its extent in terms of geographical distribution and species involved, and present *YeastFinder*, a cured online database providing a collection of information on this topic.

## *Saccharomyces cerevisiae* in Natural Environments

*Saccharomyces cerevisiae* is widely used in the industry for winemaking, brewery, and bakery, as animal and human food supplement or probiotic (Palma et al., 2015), and for biofuel, flavorings, pharmaceuticals, and enzymes production (e.g., invertases, lactases) (Parapouli et al., 2020). The physiology and genetics of this yeast have been studied in depth, as well as molecular mechanisms shared with other eukaryotes (Resnick and Cox, 2000). Despite this broad range of applications, the natural diffusion and evolution of this yeast remained unexplored until recently. The ability to overgrow other microorganisms in fermentable substrates and the widespread use of *S. cerevisiae* in the industry of fermented products lead to the hypothesis that this yeast has been domesticated and is confined to human activities (Gallone et al., 2016). However, over the last few decades, this hypothesis was compromised by the isolation of strains from natural environments (e.g., soil, barks, and water) and by the observation of the existence of strains genetically different from those used in the industry (Liti, 2015). A new hypothesis was proposed: a neutral model in which *S. cerevisiae* is functionally adapted to a range of different environments (Goddard and Greig, 2015). Despite improving our understanding of the natural spread of the budding yeast, these new findings did not clarify (i) how the yeast can survive when nutrient sources are lacking, (ii) where it was before humans started using it to ferment food, and (iii) how can this not airborne microorganism move among different environmental sources. The identification of the association between the budding yeast and social wasps helped fill in several of the gaps in our knowledge on *S. cerevisiae* natural cycle. Previous studies have already shown some aspects of yeast-insect associations (e.g., Stefanini, 2018 and Blackwell, 2017). This review recapitulates the current knowledge exclusively on associations involving *S. cerevisiae* also addressing new aspects: the extent and geographical distribution.

## *Saccharomyces cerevisiae*-Insects Association: the Yeast Side of the Coin

Social wasps and hornets are omnivorous insects that move around a broad foraging area and visit substrates that can be colonized by *S. cerevisiae* strains. Among the substrates visited by wasps, grape skin is the main source of environmental *S. cerevisiae* strains, even if this yeast represents only a minor component of the residing microbial communities. *Polistes dominula* (social wasps) and *Vespa crabro* (hornets) host in their intestines *S. cerevisiae* cells all year long, thus providing an environment in which yeasts can reside and survive in the seasonal period with less access to sugary sources (Stefanini et al., 2012). Insects can spread *S. cerevisiae* cells among environmental substrates and, also, share them within the colony, passing it among adults and to larvae (Stefanini et al., 2012). Thus, the capability of spreading yeast cells increases exponentially with the increase of the insect colony. This has pivotal importance for the diffusion of *S. cerevisiae* cells in the wild, especially considering that the increase in the demographic rate of the wasp colony occurs at the same time of grape ripening (Stefanini et al., 2012). Hence, the high frequency of *S. cerevisiae* cells isolated after the ripening period correlates these insects to the dispersion of yeasts in the vineyard (Stefanini et al., 2012). In addition to social wasps and hornets, other insects bear and spread *S. cerevisiae* cells, as proven in laboratory conditions for *Drosophila* spp. (Christiaens et al., 2014) and confirmed in the wild for bees. In fact, *S. cerevisiae* strains isolated from vineyard specimens are highly similar to strains isolated from bees caught in the same geographic region, suggesting that insects are responsible for the local dispersion of yeast cells (Goddard et al., 2010). Interestingly, the genetic and phenotypic diversity of *S. cerevisiae* does not affect the capability of different yeast strains to survive in the insect intestines (Dapporto et al., 2016; Ramazzotti et al., 2019) or to produce volatile metabolites attracting insects (Palanca et al., 2013). Hence, the whole genetic and phenotypic variability of *S. cerevisiae* can potentially attract and be vectored among natural specimens thanks to insects (Stefanini et al., 2012; Dapporto et al., 2016).

**FIGURE 1:**
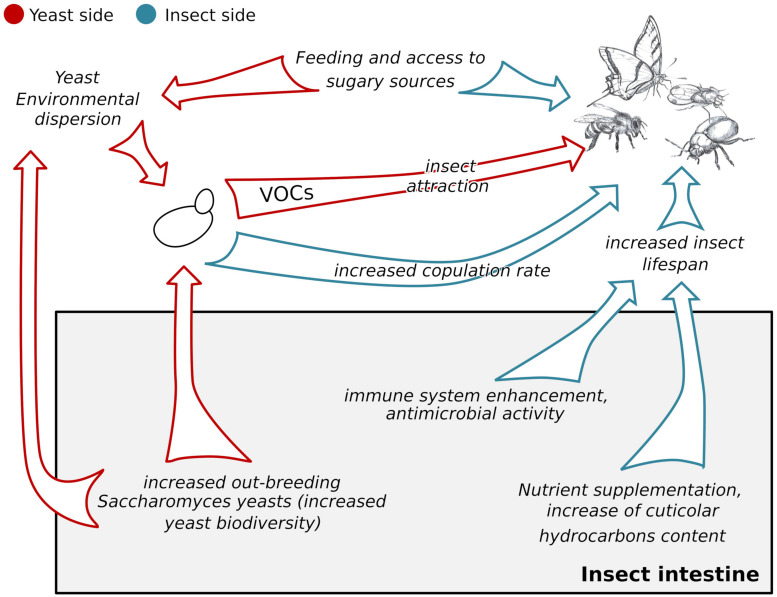
Schematic representation of the impacts of the *S. cerevisiae*-insects association on the yeast and on the insect. Red arrows indicate the impacts of the association on yeasts, blue arrows indicate the impacts of the association on insects. The gray box indicates effects of the association occurring within the insect intestines, both on *S. cerevisiae* and on the insects. VOCs = Volatile Organic Compounds.

*Saccharomyces cerevisiae* mating is infrequent in nature, possibly because wild yeast cells are mainly diploid and hence need to face sporulation and germination to be able to mate with other strains, conditions that rarely occur in nature (Cubillos et al., 2009). Contrarily, yeast mating does occur within insect intestines (Stefanini et al., 2016; Figure 1). *S. cerevisiae* spores can survive in the intestinal tract of *Drosophila melanogaster*, and, by passing through the insect intestines, the sporal ascus is broken and hence the mating among yeast strains is facilitated (Reuter et al., 2007). The differences among chemical and physiological characteristics of different tracts of the insect intestine could offer a series of environmental changes (Engel and Moran, 2013) promoting yeast sporulation and germination, and hence mating, as shown by experiments carried out in the laboratory (Stefanini et al., 2016). Hence, the intestine not only promotes the yeast ascus break, but also diploid yeast cells sporulation and yeast spores germination, and thus allowing the mating among potentially any yeast strain and ploidy (Stefanini et al., 2016).

## *Saccharomyces cerevisiae*-Insects Association: the Insect Side of the Coin

The *S. cerevisiae*-insect association has beneficial effects not only on the yeast, but also on its counterpart: insects (Figure 1).

The capability to detect food is fundamental for insect survival to obtain the nourishment and find an environment suitable for oviposition. *S. cerevisiae* attracts various insect species including *D. melanogaster* (Becher et al., 2012), *Vespula germanica*, and *V. vulgaris* (Babcock et al., 2017) to food, which is otherwise less appealing. The main features making this yeast capable of enticing insects are the presence of functional mitochondria (Schiabor et al., 2014) and the capability of producing volatile compounds, such as isoamyl acetate and ethyl acetate (Christiaens et al., 2014), which are produced at high levels by yeasts isolated from wasp intestines (Dapporto et al., 2016). Overall, these findings show that the presence of fermenting *S. cerevisiae* cells is a strong cue used by insects to detect sugary substrates. It is fair to consider that, besides *S. cerevisiae*, other microorganisms present on sugary substrates, such as *Hanseniaspora* spp. and *Gluconobacter* spp. (Bueno et al., 2019), and vectored by insects (Palanca et al., 2013; Quan and Eisen, 2018) could produce aromas attracting insects. Interestingly, species-specific attractions have been observed. While *D. melanogaster* is attracted by *S. cerevisiae*, *D. simulans* is indifferent to this yeast species (Gunther et al., 2019). At the same time, *D. melanogaster* and the subgenus *Sophophora* are preferentially attracted to baits seeded with *Hanseniaspora uvarum* than to *S. cerevisiae* and forest-dwelling *Drosophila* species (e.g., *D. tripunctata* and the *guarani* group) are more attracted by *S. cerevisiae* than by *H. uvarum* (Batista et al., 2017). The differential preferences of *Drosophila* spp. towards different yeast species may mirror what happens in the yeast–insect–morning glory ecosystem, where beetles and yeasts (mostly *Metschnikowia* spp. and *Candida* spp.) reciprocally influence the occupancy of associations-specific niches (Stefanini, 2018).

In the laboratory, insect rearing is based on the use of media providing the full range of nutrients to support larval development but also appealing to female adults and promoting egg deposition (Piper, 2017). To this aim, it is a common practice to use media including *S. cerevisiae* (Becher et al., 2012; Grangeteau et al., 2018). In fact, the yeast supports larval development mostly by providing nicotinic acid, vitamin B, pantothenic acid, inositol, choline, beta-alanine, and pimelic acid (Tatum, 1941). The presence of *S. cerevisiae* in the larval diet also defines the fitness and behavior of juvenile and adult insects (Grangeteau et al., 2018). Providing live *S. cerevisiae* cells to *Drosophila* larvae improves the copulation rate, increases the cuticular hydrocarbon content, extends the insect life, and makes adults preferring food supplemented with the yeast, compared to a diet based on yeast extracts or lacking the yeast (Grangeteau et al., 2018; Murgier et al., 2019). Transcriptional analyses carried out on *D. melanogaster* adults developed from germ-free larvae showed the over-expression of genes involved in several metabolic pathways, if the insect diet was supplemented with live *S. cerevisiae* compared to single or multiple bacterial species (Elya et al., 2016). Despite this effect being observed only in intestinal cells and not at the whole-body level, hence suggesting the impact of yeast on insects is only local, it has to be considered that over the developmental process genes expression is rapidly regulated by multiple factors. Thus, time-course transcriptional analyses would be required to appreciate at the molecular level the impact of *S. cerevisiae* on insect development. The impact of *S. cerevisiae* on development and related traits varies according to the yeast species. For instance, even if a substrate supplemented with the budding yeast improves the survival of adult mosquitoes (*Culex pipiens*) compared to substrates supplemented with other yeast species, it is not preferred for oviposition by gravid *C. pipiens* females (Díaz-Nieto et al., 2016).

As in bigger animals, even in insects the fate and impact of the encountered microorganisms are often determined by the host immune system. The insect immune system includes a cellular and a humoural component (Lemaitre and Hoffmann, 2007). Although several receptors on immune cells have been described as responsible for the recognition of microorganisms, their role in the response to *S. cerevisiae* and yeasts in general is still unclear (Lu and St Leger, 2016). Conversely, an entire pathway of the humoral response is responsible for the insect’s reaction to yeasts: the Toll signaling pathway (Roh et al., 2009). When triggered by yeast cell wall *β*-glucans and proteases, this pathway induces the expression of Drosomycin, an antimicrobial agent (Gottar et al., 2006). Insects can also fight potential yeast pathogens through the Duox response pathway, which induces the production of not-specific antimicrobial reactive oxygen species (Hoang et al., 2015). To note, the vast majority of information on the insect immune response to *S. cerevisiae* has been obtained by using the *Drosophila* spp. model that presents perturbations in the Toll pathway (Alarco et al., 2004), making this insect susceptible to the budding yeast (Lionakis, 2011). Alternatively, the use of *Galleria mellonella*, naturally susceptible to *S. cerevisiae*, yielded fundamental information that could not be gathered with fruit flies. For instance, *G. mellonella* provided the first insights on the capability of a pre-exposure to *S. cerevisiae* cells or glucans to protect the insect against a subsequent infection with a lethal dose of *Candida albicans* (Bergin et al., 2006). This immune-enhancing elicited by *S. cerevisiae* has been recently confirmed in the social wasps *Polistes dominula*, which become more resistant to *Escherichia coli* infections upon pre-immunization with the yeast (Meriggi et al., 2019).

## Biogeography and Diffusion

**FIGURE 2:**
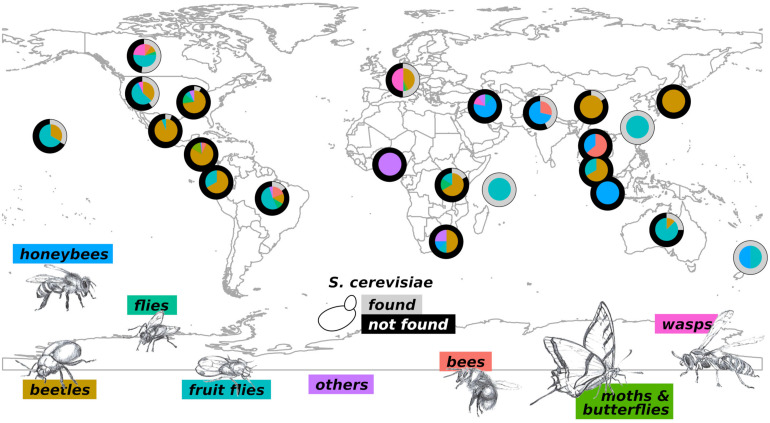
Biogeography and extent of the *S. cerevisiae*-insect association. For each geographical location, the outer ring of the pie chart shows the percentages of insects in which *S. cerevisiae* has been (gray) or has not been (black) identified. The inner parts of pie charts show the types of insects investigated in the corresponding area and the relative proportions; colors are indicated for each insect group at the bottom of the figure. The group “others” refers to insects poorly represented: ants, lacewings, termites, mites, and mosquitoes.

Insects can colonize habitats with extremely different characteristics and are considered, as defined by E.O. Wilson, “*the little things that run the world*” (Wilson, 1987). The large number of insect species makes it impossible to comprehensively analyze their biodiversity. Of the estimated 6 million species, only 1 million are known (Larsen et al., 2017). For a matter of clarity, we will report here information on insects broadly grouped. We have, however, created a detailed on-line database that can be browsed by the reader (YeastFinder,^1^). *S. cerevisiae* has been identified, through isolation or metabarcoding, in the intestine or on the body of several insects all over the World (Figure 2). Notably, studies carried out so far lack consistency in the methods adopted for yeast isolation, and this may greatly impact the capability of identifying *S. cerevisiae* associated with insects. However, in this review we will neglect the heterogeneity of the adopted methods, leaving the exploration of this topic to dedicated future studies. Interestingly, *S. cerevisiae* has not been found in insects caught in South Africa (SAf), Ecuador, Thailand, Indonesia, Nigeria (the only study on termites), Iran, Japan (J), Malaysia, and Central America (CA: Panama, Costa Rica, and Guatemala). It is worth to consider that the investigations carried out in Ecuador and Malaysia (Freitas et al., 2013), Thailand (Saksinchai et al., 2015), Indonesia (Basukriadi et al., 2010), Nigeria (Adelabu et al., 2019), and Iran (Siavoshi et al., 2018) are related to individual studies, hence the lack of identification of *S. cerevisiae* could be ascribed to the procedure adopted for yeast isolation. On the other hand, multiple studies failed in identifying *S. cerevisiae* in association with beetles, honeybees and mosquitoes, butterflies, mites, and moths collected in J (Toki et al., 2012; Ninomiya et al., 2013), SAf (de Vega et al., 2012; Steyn et al., 2016), and CA (Lachance et al., 2001a, b, 2006, Suh and Blackwell, 2006; Suh et al., 2006, 2007; Rivera et al., 2009; Urbina et al., 2013; Ravenscraft et al., 2018). The lack of identification of *S. cerevisiae* in insects of these areas could indicate an unusual situation that is worth to be further investigated. All the insects investigated in New Zealand, Taiwan, and Seychelles Islands bore *S. cerevisiae* (Figure 2). However, the number of cases studied in these areas is low (*n* = 6), and hence this observation may be poorly representative of the real situation.

Multiple studies investigating various insects in other locations (shown in Figure 2) indicated an even geographical distribution of *S. cerevisiae*. The budding yeast has not been isolated from ants, lacewings, termites, mites, and mosquitoes (the group “others” in Figure 2), which were, however, poorly investigated (two species in different locations at most) (Carreiro et al., 1997; Lachance et al., 2003; Suh et al., 2005; Nguyen et al., 2006; Steyn et al., 2016; Siavoshi et al., 2018). Similarly, *S. cerevisiae* has not been found in butterflies (*n* = 11 species in different locations) and moths (*n* = 4) (Suh et al., 2006; Witzgall et al., 2012; Ravenscraft et al., 2018). Interestingly, only the 0.03% of beetles, which have been widely investigated (*n* = 236), bear *S. cerevisiae* (Kurtzman and Robnett, 1998; Lachance et al., 2001a, b, 2006; Six, 2003; Suh and Blackwell, 2004, Suh et al., 2005, 2006, 2007, 2013; Delalibera et al., 2005; Nguyen et al., 2006; Rosa et al., 2007; Rivera et al., 2009; de Vega et al., 2012; Hui et al., 2012; Toki et al., 2012; Freitas et al., 2013; Kaltenpoth and Steiger, 2013; Ninomiya et al., 2013; Urbina et al., 2013; Cline et al., 2014; Ren et al., 2014, 2015; Liu et al., 2016; Tanahashi and Hawes, 2016; Wang et al., 2016; Briones-Roblero et al., 2017; Chai et al., 2019). Similarly, bees only accidentally bear *S. cerevisiae*, with only 1 occurrence over 21 reported cases (Sandhu and Waraich, 1985; Lachance et al., 2003; Rosa et al., 2003; Daniel et al., 2013; Charron et al., 2014; Saksinchai et al., 2015). Conversely, *S. cerevisiae* has been found in a large portion of investigated flies, fruit flies, honey-bees, and wasps (29, 57, 20, and 71%, respectively) (Phaff and Knapp, 1956; Batra et al., 1973; Sandhu and Waraich, 1985; Morais et al., 1993, 1994; Rosa et al., 1994; Lachance et al., 1995, 2003, 2006; Suh et al., 2005; Nguyen et al., 2006, 2007; Basukriadi et al., 2010; Goddard et al., 2010; Chandler et al., 2012; de Vega et al., 2012; Hamby et al., 2012; Stefanini et al., 2012; Freitas et al., 2013; Buser et al., 2014; Charron et al., 2014; Lam and Howell, 2015; Saksinchai et al., 2015; Batista et al., 2017; Deutscher et al., 2017; Jimenez et al., 2017; Piper et al., 2017; Quan and Eisen, 2018; Siavoshi et al., 2018; dos Santos et al., 2019; Meriggi et al., 2019; Park et al., 2019). A few possible scenarios could explain the higher occurrence of *S. cerevisiae* in these groups of insects: (i) they are more prone to visit human-related environments, such as wineries and vineyards, that are likely to host higher amounts of *S. cerevisiae* cells, (ii) they are more attracted by substrates inhabited by the budding yeast compared to other insects, (iii) diet and physical-chemical intestine conditions facilitate the housing of *S. cerevisiae*.

## Conclusion and Perspectives

According to the reports gathered for this review, it appears that every group of insects can bear *S. cerevisiae*, but only further and more detailed studies investigating a higher number of a broader range of insect species, as well as the standardization of isolation and identification methodologies, will consolidate this observation. Also, further studies on the geographical extent of this phenomenon would allow evaluating the existence of different physiological characteristics among insect species that favor or prevent the instauration of the association with the budding yeast. Aiming at this, it will be fundamental to also include groups neglected so far. For instance, planthoppers, mosquitoes, and spiders have not or have only poorly been investigated, albeit they could represent an unprecedented source of information as they visit and forage on a broad range of environmental sources. In addition, *S. cerevisiae* has been shown to have an impact on spiders’ behavior and health (Tietjen et al., 1987; Patt et al., 2012), and, especially considering that spiders are mostly carnivores, exploring this association would provide insightful information on the role of this yeast in prey hunting and interactions among species.

Overall, the reports published so far depict a tangled relationship between insect and yeast, in which various factors define the insect attraction to yeasts and the impact of this yeast on insect health. Understanding the factors responsible for the attraction of insects by yeasts, also by further exploring the differences among insect species has also important applications. A better understanding of the factors regulating this complex field will provide relevant information potentially useful to ideate approaches to use *S. cerevisiae* as a promoter of insect health or as a pest control. For instance, dissecting the capability of enhancing the host immune reaction against pathogenic microorganisms would be very useful in the fight against the worldwide decline of honeybees and pollinators (Wagner, 2020).

Only further studies will allow us to fully unravel the influence of *S. cerevisiae* on insects, and the potential applications of strains isolated from this natural source.

## Author Contributions

IS ideated the review. NM and IS gathered the data. DC, MD, NM, and IS wrote the manuscript. All authors contributed to the article and approved the submitted version.

## Conflict of Interest

The authors declare that the research was conducted in the absence of any commercial or financial relationships that could be construed as a potential conflict of interest.

## Footnotes

1 www.stefaninilab.com/tools/
